# Correlation Between the SARA and A-T NEST Clinical Severity Scores in Adults with Ataxia-Telangiectasia

**DOI:** 10.1007/s12311-023-01528-2

**Published:** 2023-04-10

**Authors:** Toby Major, May Yung Tiet, Rita Horvath, Anke E. Hensiek

**Affiliations:** 1 School of Clinical Medicine, University of Cambridge, Hills Road, Cambridge, CB2 0SP UK; 2 Department of Clinical Neurosciences, University of Cambridge, Cambridge, CB2 2QQ UK; 3 Department of Neurology, Cambridge University Hospitals NHS Foundation Trust, Cambridge, CB2 0QQ UK

**Keywords:** Ataxia telangiectasia, Cerebellar ataxia, Severity of illness index

## Abstract

Ataxia-Telangiectasia (A-T) is an autosomal recessive neurodegenerative disease associated with cerebellar ataxia and extrapyramidal features. A-T has a complex and diverse phenotype with varying rates of disease progression. The development of robust natural history studies and therapeutic trials relies on the accurate recording of phenotype using relevant and validated severity of illness indexes. We compared the commonly used Scale for the Assessment and Rating of Ataxia (SARA) and the disease-specific A-T Neurological Examination Scale Toolkit (A-T NEST), in our adult A-T cohort. We found a strong correlation between A-T NEST and the established SARA score, validating the use of A-T NEST and SARA in capturing the natural history of A-T patients.

**Supplementary Information:**

The online version contains supplementary material available at 10.1007/s12311-023-01528-2.

## Introduction

### Ataxia-Telangiectasia

Ataxia-Telangiectasia (A-T) is a rare autosomal recessive neurodegenerative disease characterised by a constellation of neurological symptoms including cerebellar ataxia and extrapyramidal features. A-T is also associated with immunodeficiency, malignancy and other systemic complications [1]. The disease arises due to mutations in the Ataxia Telangiectasia Mutated (*ATM*) gene, which encodes a serine-threonine protein kinase that is heavily implicated in the cellular response to double-stranded DNA damage [2].

The complex nature of A-T and the significant phenotypic variation between individuals can be in part explained by the classification of two distinct forms of the disease. Classic (typical) A-T is considered clinically more severe and is associated with a complete loss of ATM kinase function. Meanwhile, variant (atypical) A-T causes a milder phenotype, consisting of neurological symptoms and cancer susceptibility, often without systemic illness, owed to residual ATM kinase function [3].

The heterogeneity of A-T phenotypes, alongside the varying rates of disease progression, limits the development of robust natural history studies and therapeutic trials to effectively assess treatment response. Whilst established ataxia disease scoring systems, such as the Scale for the Assessment and Rating of Ataxia (SARA), are used in clinical practice, patients with A-T often have additional extrapyramidal symptoms which are not captured by this scoring system. In recent years, disease-specific severity of illness indexes, such as the A-T Neurological Examination Scale Toolkit (A-T NEST), have been developed to address the clinical heterogeneity of A-T and stratify disease severity in clinical trials and epidemiological studies.

### SARA

The SARA score was first proposed as a measure of ataxia symptom severity in patients with spinocerebellar ataxia (SCA) [4]. It assesses 8 domains: gait, stance, sitting ability, speech disturbance, finger chase, nose-finger test, fast alternating hand movements and heel-shin slide. It carries a maximum score of 40 with higher scores indicating more severe ataxia symptoms [4]. SARA was initially validated in SCA patients in two clinical trials where it was found to correlate with the Barthel Index and part IV of the Unified Huntington’s Disease Rating Scale [4]. Test–retest and interrater reliability, as well as internal consistency, have been demonstrated to be high [4] and the time taken to assess patients using the SARA score is considered less than other ataxia scoring systems [5].

However, the SARA score remains unvalidated specifically for use in A-T and is not without its limitations. The initial studies noted that the score did not consider symptoms that manifest alongside the ataxia in patients with SCA [4]. A-T patients often have extrapyramidal symptoms which can cause significant morbidity. We have previously demonstrated that some variant A-T patients have predominant extrapyramidal symptoms or peripheral neuropathy rather than ataxia [6].

To date, there have been few clinical trials utilising SARA in patients with A-T [7, 8] and none directly validating its use in this disease. The weighting of the total score is also disproportionately influenced by the gait domain. Many classical A-T patients lose ambulation by their teenage years, and it is unclear how this influences the assessment of disease progression in non-ambulant patients who continue to report clinical deterioration [9]. Finally, although the original paper reported no significant score ceiling effect amongst the patient cohort, there have since been accounts of this affecting both the total score and particular domains [10].

### A-T NEST

The A-T NEST was developed from the A-T Index (Crawford Score) first proposed in 2000 [11] and, in its current form, assesses 53 items across 6 core neurological domains. The domains assessed include communication ability, eye movements, ataxia, movement disorder, muscle power and the presence of neuropathy. The inclusion of domains assessing extrapyramidal dysfunction and eye movement involvement is particularly pertinent given that A-T can present with these as the predominant or only clinical features. An additional 11 items assessed over 4 domains focussing on growth, nutrition, cognition and mental state make up the neurological-related component of the A-T NEST. These additional domains are also not assessed by the SARA score. The core neurological domains summate to generate a pure neurological A-T NEST score out of a total of 100 with individuals scoring lower the more severe their A-T symptoms.

Linear regression analysis showed that the A-T NEST score correlated strongly with age which, with the assumption of the progressive nature of A-T, suggests that the score is useful for longitudinal tracking of A-T symptoms. In addition, interrater variation was low suggesting minimal subjective influence [11]. One longitudinal study involving children and young people with A-T demonstrated a positive correlation between the revised A-T NEST score and the original A-T Index [12]. However, neither the A-T NEST nor the original A-T Index have been appropriately validated for use in A-T patients by means of comparing against well-established and validated scoring systems. Despite this, the A-T NEST has continued to be used as a means of tracking the longitudinal progression of patient symptoms [13]. A-T NEST provides valuable information about extrapyramidal symptoms and oculomotor issues, frequently encountered in A-T patients. In practice, the A-T NEST score takes significantly longer to assess compared to the SARA score. The SARA score has also been used in previous interventional studies in A-T [14, 15] but does not evaluate extrapyramidal and eye movements.

This retrospective longitudinal study assesses the application of the A-T NEST score in an adult A-T patient cohort and investigates the longitudinal relationship between the A-T NEST and SARA scores.

## Materials and Methods

The medical records of 55 individuals (males = 27, females = 28) with genetically confirmed diagnoses of A-T who attended the National Adult A-T Clinic at Royal Papworth Hospital over a 7-year period were retrospectively reviewed in a longitudinal manner. Of these, 28 patients had classic A-T and the remaining 27 patients had variant A-T (Table 1). The duration of time over which the patients were assessed ranged from 0 to 5 years on an annual or biannual basis. SARA and A-T NEST scores were extracted from these records generating a total of 74 instances where both the SARA and A-T NEST scores were recorded during the same clinical encounter.

**Table 1:** Demographics of patients assessed, including the type of A-T diagnosed (classic vs variant), the sex of the patient as well as the age at which they were first assessed

Characteristic	Classic A-T (*n* = 28)	Variant A-T (*n* = 27)
Sex—number (%)		
Male	16 (57%)	11 (41%)
Female	12 (43%)	16 (59%)
Age in years—mean ± SD (range)		
At first assessment	21.2 ± 7.1 (16–42)	35.9 ± 12.7 (16–55)

Simple linear regression modelling was then used to assess if SARA scores accurately predicted the A-T NEST scores obtained during the same clinical encounter. This was conducted in both the classic and variant A-T patient groups in isolation as well as in the pooled cohort consisting of both groups. Spearman’s rank correlation was then used to assess the relationship between SARA and A-T NEST scores in these groups to determine if any such correlation was evident longitudinally. A cross-sectional analysis was also performed for all patient visits in a single year. Statistical and graphical analysis was conducted in GraphPad Prism version 9.

## Results

**Fig. 1:**
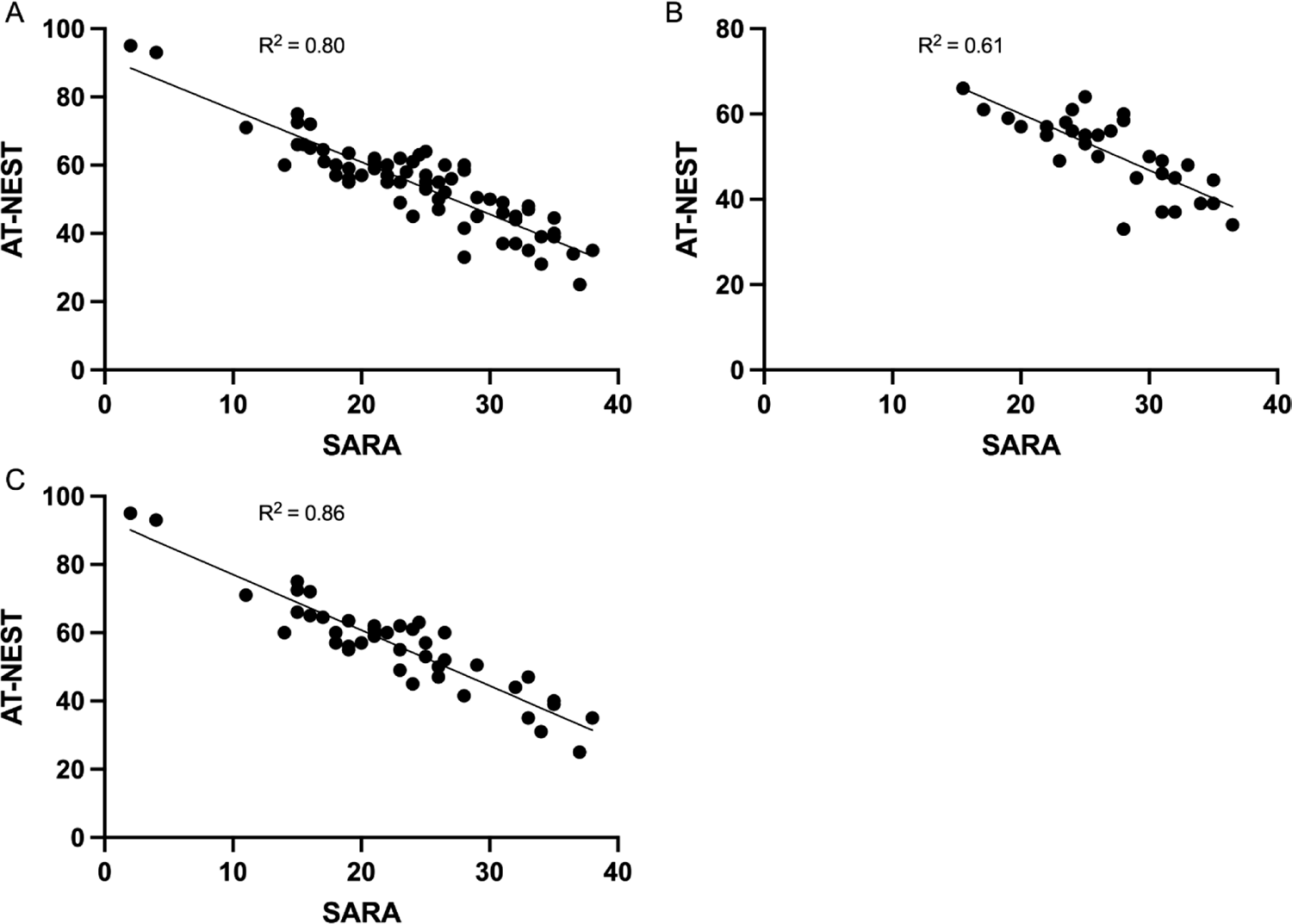
Correlation between A-T NEST and SARA. Scatter plots comparing total A-T NEST and SARA scores calculated during the same clinical encounter for **A** the whole A-T patient cohort (*R*^2^ = 0.80, *F*_(1,72)_ = 292.8, *p* < 0.0001), **B** the classic A-T patient cohort (*R*^2^ = 0.61, *F*_(1,30)_ = 46.5, *p* < 0.0001) and **C** the variant A-T patient cohort (*R*^2^ = 0.86, *F*_(1,41)_ = 243.9, *p* < 0.0001). The coefficient of determination (*R*^2^) denoting the goodness of fit of the linear models is displayed

The linear models produced by regression analysis showed that SARA scores accurately predicted A-T NEST scores. This was evident in the whole A-T patient cohort (Fig. 1A) and in the classic (Fig. 1B) and variant (Fig. 1C) sub-cohorts. The regressions were all statistically significant.

Spearman rank correlation showed a significant correlation between AT-NEST and SARA scores in the whole A-T patient cohort (*r*_(72)_ =  − 0.86, 95% CI [− 0.78, − 0.91], *p* < 0.0001) as well as in the classical (*r*_(30)_ =  − 0.79, 95% CI [− 0.89, − 0.60], *p* < 0.0001) and variant A-T sub-groups (*r*_(41)_ =  − 0.87, 95% CI [− 0.93, − 0.77], *p* < 0.0001).

## Discussion

This study aims to validate the A-T NEST by comparing it with a well-established severity of illness index used in patients with A-T. A strong correlation between both scores has been found at the single time point and longitudinal assessment level both in patients with classic and variant A-T. This work has shown the A-T NEST to be a valid means of measuring disease progression in adult patients with A-T.

The main limitations of this study include the reliance on paper clinical notes and the retrospective analysis. Some data points were missing but our large cohort has enabled a comprehensive assessment of the validity of A-T NEST and SARA scores in recording the natural history of A-T. A further limitation is evaluating the appropriateness of only two examination tools. The strength of our study is the diverse clinical phenotype of our cohort, including patients with predominant extrapyramidal phenotypes. Our study confirms that SARA and A-T NEST remain useful tools in capturing disease progression. Future studies would benefit from a prospective analysis of the effect of disease duration and age in various A-T phenotypes using a diverse range of clinical evaluation tools.

## Conclusion

The A-T NEST is a valid severity of illness index for use in adult patients with classic and variant A-T. The A-T NEST correlates strongly with the SARA score at the single timepoint level and changes proportionally with the SARA score longitudinally which demonstrates its ability to track the severity of the disease over time.

### Supplementary Information

Below is the link to the electronic supplementary material.

Supplementary file1 (DOCX 35 KB)

## Data Availability

The data collected forms part of the Genotype and Phenotype study. Interested persons may contact the corresponding author, Dr Anke Hensiek, for further details about the study data.
