# Supplementary material for: Correlation Between the SARA and A-T NEST Clinical Severity Scores in Adults with Ataxia-Telangiectasia
Source: Cerebellum. 2023 Apr 10;23(2):455–8. doi: 10.1007/s12311-023-01528-2 (PMC10951025; doi:10.1007/s12311-023-01528-2)
Supplement: Supplementary file 1 — Supplementary file1 (DOCX 35 KB) [file 12311_2023_1528_MOESM1_ESM.docx]

Supplementary Information

Supplementary Figure 1: Correlation between A-T NEST and SARA cross-sectional analysis. Scatter plots comparing total A-T NEST and SARA scores calculated during the same clinical encounter for the whole A-T patient cohort in one year (R^2^ = 0.82, F_(1,21)_ = 41.96, p < 0.0001)
